
JOURNAL INFORMATION
==============================
NLM Title Abbreviation: Wirtschaftsdienst
Journal ID: Wirtschaftsdienst
NLM Journal ID: 101086612

Wirtschaftsdienst (Hamburg, Germany : 1949)

ISSN: 0043-6275

ARTICLE INFORMATION
==============================
PMCID: PMC7174820
PMID: 32336798
DOI: 10.1007/s10273-020-2624-4
Article ID: 2624
Article version: 1
Subjects: Zeitgespräch

Steigende Einkommen, sinkende Sorgen — die Zeit vor Corona

Niehues Judith niehues@iwkoeln.de

Stockhausen Maximilian stockhausen@iwkoeln.de

grid.473597.b 0000 0000 9854 4639 Methodenentwicklung, Institut der deutschen Wirtschaft Köln e.V., Konrad-Adenauer-Ufer 21, 50668 Köln, Deutschland
Electronic publication date: 2020 Apr 22
Print publication date: 2020
Volume: 100
Issue: 4
First page: 237
Last page: 241
Copyright: © Der/die Autor(en) 2020
License: Open Access Dieser Artikel wird unter der Creative Commons Namensnennung 4.0 International Lizenz veröffentlicht, welche die Nutzung, Vervielfältigung, Bearbeitung, Verbreitung und Wiedergabe in jeglichem Medium und Format erlaubt, sofern Sie den/die ursprünglichen Autor(en) und die Quelle ordnungsgemäß nennen, einen Link zur Creative Commons Lizenz beifügen und angeben, ob Änderungen vorgenommen wurden.
Die in diesem Artikel enthaltenen Bilder und sonstiges Drittmaterial unterliegen ebenfalls der genannten Creative Commons Lizenz, sofern sich aus der Abbildungslegende nichts anderes ergibt. Sofern das betreffende Material nicht unter der genannten Creative Commons Lizenz steht und die betreffende Handlung nicht nach gesetzlichen Vorschriften erlaubt ist, ist für die oben aufgeführten Weiterverwendungen des Materials die Einwilligung des jeweiligen Rechteinhabers einzuholen.
Weitere Details zur Lizenz entnehmen Sie bitte der Lizenzinformation auf http://creativecommons.org/licenses/by/4.0/deed.de.
License URL: https://creativecommons.org/licenses/by/4.0/

issue-copyright-statement: © The Author(s) 2020

==============================

Das Coronavirus erschüttert die Welt. Das gesellschaftliche und wirtschaftliche Leben, wie wir es bisher gekannt haben, ist zum Erliegen gekommen und die Unsicherheit über die Zukunft ist groß. Niemand kann vorhersagen, wie lange die Krise anhalten wird. Klar ist jedoch, dass sie mit längerer Dauer einen zunehmend größeren volkswirtschaftlichen Schaden anrichten wird. Die Verteilungswirkungen der derzeitigen Entwicklungen hängen von zahlreichen Faktoren ab und lassen sich zum jetzigen Zeitpunkt kaum prognostizieren. Zudem liegen der Wissenschaft keine hinreichend aktuellen Einkommens- und Vermögensdaten vor, die eine gegenwärtige Bewertung erlauben würden. Daher fokussiert dieser Beitrag auf Auswertungen verfügbarer Verteilungsdaten, die bis zum Jahr 2017 reichen.

Für die Zeit vor der Corona-Krise lässt sich das gesamtwirtschaftliche Umfeld als sehr positiv beschreiben. Trotz des Einbruchs während der Finanz- und Wirtschaftskrise betrug das jahresdurchschnittliche inflationsbereinigte Wachstum des Bruttoinlandsprodukts (BIP) zwischen 2005 und 2017 rund 1,6 %. Seit ihrem Höhepunkt im Jahr 2005 mit 11,7 %, ist die Arbeitslosenquote auf zuletzt 5 % im Jahr 2019 gesunken (Bundesagentur für Arbeit [BA], 2019) – einem Tiefststand seit der Wiedervereinigung. Begleitet wurde diese Tendenz von einer positiven Reallohnentwicklung, die sich auch in einer gestiegenen Lohnquote auf gesamtwirtschaftlicher Ebene widerspiegelt – zuletzt lag diese 2019 mit rund 72 % wieder auf dem Niveau der 1990er Jahre (Statistisches Bundesamt, 2020).

## Einkommenszuwächse in allen Schichten

Der alleinige Blick auf die allgemeine Erwerbs- und Lohnentwicklung reicht aber nicht aus, um Aussagen über die finanzielle Situation der Haushalte zu treffen. Veränderte Lebensformen, Arbeitszeitanpassungen, differenzierte Lohnentwicklungen oder staatliche Eingriffe zur Umverteilung haben einen erheblichen Einfluss auf die materielle Ausstattung der Haushalte und ihre Konsummöglichkeiten. Daher sollten die verfügbaren Haushaltseinkommen (oder Haushaltsnettoeinkommen) betrachtet werden.[1]

1 Die Haushaltsnettoeinkommen verstehen sich als Summe der Arbeits- und Vermögenseinkommen aller Haushaltsmitglieder zuzüglich Renten- und Transferzahlungen und abzüglich staatlicher Abgaben. Ebenso wird der Nettomietvorteil selbstgenutzten Wohneigentums berücksichtigt. Wie konventionell üblich wird zur Bedarfsgewichtung die modifizierte Skala der Organisation für wirtschaftliche Zusammenarbeit und Entwicklung (OECD) herangezogen.

In der Debatte um die Entwicklung der Einkommensungleichheit besteht weitestgehend Einigkeit darin, dass die Nettoeinkommen heute ungleicher verteilt sind als beispielsweise in den 1980er oder 1990er Jahren. Strittig ist hingegen die Deutung der Entwicklung ab 2005, die daher im Fokus der folgenden Ausführungen stehen soll.[2]

Mithilfe der Daten des Sozio-oekonomischen Panels (SOEP) lässt sich die Entwicklung der Einkommen in verschiedenen Einkommensgruppen analysieren. Es zeigt sich, dass im Durchschnitt von 2005 bis 2017 alle Einkommensgruppen reale Zuwächse verbuchen konnten (vgl. Abbildung 1). Dies gilt in besonderem Maße für die Einkommensmitte, hier repräsentiert durch das 5. Einkommensdezil, die ihr Einkommen seit 2013 besonders stark steigern konnte. Auch die unteren 10 % konnten gegenüber 2005 im Durchschnitt reale Zugewinne verzeichnen. Die Ergebnisse stehen in einem Widerspruch zu vorherigen Befunden, dass die Gruppe der ärmsten 10 % gegenüber 2005 reale Einkommensverluste hinnehmen musste (Spannagel und Molitor, 2019, 12 f.).

Die unterschiedliche Bewertung kommt im Wesentlichen durch eine Datenrevision zustande, die im SOEP auch rückwirkend erfolgt.[3] Mit der aktuell verfügbaren Datenlieferung 2018 (SOEP v35) wurde aus einem durchschnittlichen Rückgang der Realeinkommen der unteren 10 % zwischen 2005 und 2016 um rund 2 Prozentpunkte ein Zuwachs in Höhe von 3 Prozentpunkten. Grundsätzlich ist bei dieser Art von Querschnittsbetrachtungen zu betonen, dass sich die Einkommensgruppen in den unterschiedlichen Jahren keineswegs zwangsläufig aus den jeweils gleichen Personen zusammensetzen. Bereits mit der Datenlieferung des Jahres 2017 (SOEP v34) konnte beispielsweise gezeigt werden, dass die Entwicklung der Einkommensungleichheit von 2015 bis 2016 im Wesentlichen dadurch verursacht wurde, dass im SOEP spezielle Geflüchteten-Stichproben neu mit in die Betrachtung eingeflossen sind (vgl. Peichl, 2020; Abbildung 1).

Der beobachtbare durchschnittliche Einkommensrückgang ging somit nicht darauf zurück, dass gleiche Personen ärmer geworden sind, sondern dass Geflüchtete mit eher geringen Einkommen neu in die Analyse eingegangen sind. Ähnlich verhält es sich mit dem deutlichen Realeinkommensverlust der unteren 10 % zwischen 2012 und 2013, der im Wesentlichen auf die Ergänzung einer umfangreichen Migrationsstichprobe zurückgeht (Niehues, 2017).

2 Siehe Stockhausen und Calderón (2020) für eine ausführliche Diskussion der Entwicklung seit der Wiedervereinigung.

3 Eine Diskussion des Einflusses der Datenrevisionen auf die Entwicklung der Armutsrisikoquote findet sich beispielsweise in Peichl (2020, 7 f.).

Abbildung 1

**Entwicklung der verfügbaren Haushaltseinkommen seit 2005**

2005 = 100, bedarfsgewichtete Einkommen (modifizierte OECD-Skala)

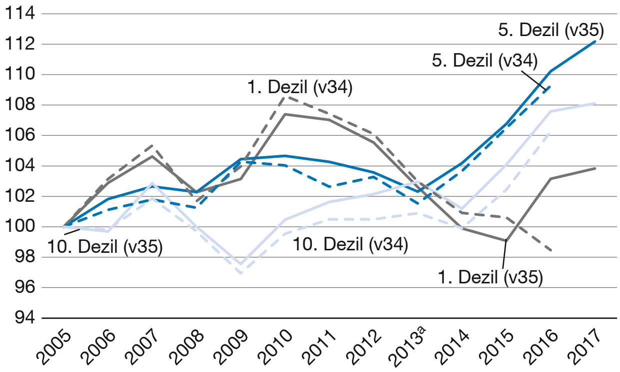

[a] Zeitreihenbruch durch Integration von Subsample M1 (Migration 1995-2011).

Quellen: SOEP (2020, v35); SOEP (2019, v34); eigene Berechnungen.

## Stabile Einkommensverteilung seit 2005

Die relativ gleichmäßige Entwicklung der Einkommen nach 2005 schlägt sich ebenfalls in einer stabilen Verteilung der verfügbaren Haushaltseinkommen nieder. Der Gini-Koeffizient schwankt seit 2005 um einen Wert von 0,29 und ist damit weitestgehend stabil geblieben. Gegenüber den 1990er Jahren liegt das Niveau des Gini-Koeffizienten jedoch höher. Nach einer Phase des leichten Rückgangs zwischen 2005 und 2009 lag das Niveau im Jahr 2013 auf einem zwischenzeitlichen Hoch, das gegenüber 2005 jedoch keine statistisch signifikante Steigerung bedeutete. Zuletzt bewegte sich der Gini-Koeffizient weitestgehend seitwärts und lag 2017 erneut bei einem Wert von rund 0,29 Punkten. Auch alternative Ungleichheitskennziffern, beispielsweise Perzentilverhältnisse, deuten nicht darauf hin, dass sich die Einkommensungleichheit in Deutschland ungebremst erhöhen würde. Im Besonderen ist darauf zu verweisen, dass sich durch die Datenrevision mit der SOEP-Welle 2018 auch die Entwicklung der Perzentilverhältnisse von 2015 bis 2016 nahezu umgekehrt hat.

Angesichts der gewachsenen Herausforderungen durch den demografischen Wandel, einem Trend zum Alleinleben oder auch den jüngsten Migrationsbewegungen ist dies ein erfreuliches Ergebnis. Die Veränderungen in der Zusammensetzung der Bevölkerung bieten auch Erklärungsansätze, warum die Einkommensverteilung angesichts der guten konjunkturellen Voraussetzungen in den

Abbildung 2

**Entwicklung der Nettovermögensungleichheit in Deutschland**

Gini-Koeffizient

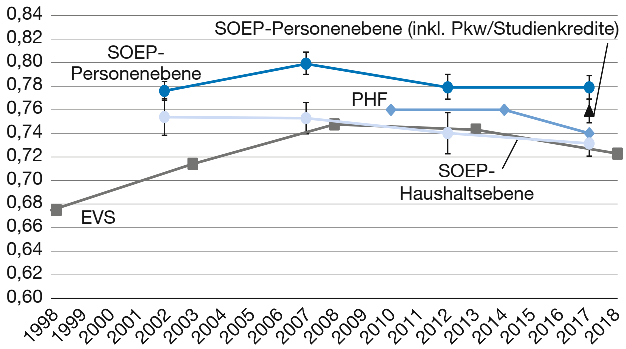

Anmerkungen: Im SOEP werden Personen ab 17 Jahren in Privathaushalten betrachtet (ohne Personen der Flüchtlingssamples M3 bis M5), Hochrechnungsfaktoren mit erster Befragungswelle. In der Einkommens- und Verbrauchsstichprobe (EVS) und in der Panelstudie „Private Haushalte und ihre Finanzen (PHF)" wird die Nettovermögensverteilung auf Haushaltsebene bestimmt. Tendenziell fällt die Ungleichheit auf der Haushaltsebene geringer aus.

Quellen: Grabka und Halbmeier (2019); eigene Berechnungen für SOEP-Haushaltsebene; Deutsche Bundesbank (2019) für PHF; Bundesministerium für Arbeit und Soziales (2020) für EVS; eigene Darstellung.

vergangenen Jahren nicht gleichmäßiger geworden ist.[4] Gleichzeitig tritt in der Diskussion zunehmend die Frage nach der Datenqualität in den Vordergrund, wenn es um die korrekte Abbildung zeitlicher Trends geht (Niehues und Stockhausen, 2020; Peichl, 2020). Zuletzt bekräftigt der Blick auf die Datenrevisionen, dass beobachtbare Veränderungen auch immer vor dem Hintergrund möglicher statistischer Unsicherheit beurteilt werden sollten.

## Keine Zunahme der Vermögenskonzentration

Eine ähnlich kontroverse Debatte wird über die Veränderung der Vermögensungleichheit in Deutschland geführt. Dabei steht oftmals die Vorstellung im Raum, dass die Vermögenskonzentration stetig zunehmen würde. Gemäß Haushaltsbefragungsdaten der Deutschen Bundesbank (2019) zeigt sich jedoch, dass der Anteil der vermögendsten 10 % am gesamten Nettovermögen von 59 % im Jahr 2010 auf 55 % im Jahr 2017 leicht gesunken ist (vgl. Abbildung 2).[5] Dabei weist die Bundesbank darauf hin, dass der Rückgang zwischen 2014 und 2017 mit Vorsicht zu interpretieren ist, da beispielsweise Betriebsvermögen schlechter abgebildet wurden.

Verwendet man die Vermögensdaten des SOEP, die seit dem Jahr 2002 alle fünf Jahre erhoben werden, so liegt die individuelle Nettovermögensungleichheit 2017 auf dem Niveau des Jahres 2002. Gegenüber 2007 ist sie sogar statistisch signifikant gesunken (bei einem Signifikanzniveau von 95 %). Es zeichnet sich somit ein ähnliches Bild ab. Auch Hinzuschätzungen am oberen Rand verändern die beobachteten Trends nicht grundlegend (Grabka und Westermeier, 2015). Entgegen der weitläufigen Wahrnehmung gibt es zumindest für den Zeitraum seit der Finanzkrise keine empirische Evidenz für eine steigende Vermögensungleichheit. Mit Blick auf die Schwierigkeiten bei der Erfassung von Hochvermögenden und der Abbildung diverser Vermögenswerte sind beobachtbare Trends hier gleichwohl mit besonderer Vorsicht zu interpretieren. Von einer unaufhörlich zunehmenden Vermögensungleichheit kann auf Grundlage der verfügbaren Datenbestände für das vergangene Jahrzehnt jedoch keine Rede sein.

Allein im Vergleich mit den 1990er Jahren zeigt sich nach Maßgabe der Haushaltsbefragungsdaten der Einkommens- und Verbrauchsstichprobe (EVS) eine Steigerung der Nettovermögensungleichheit. Die EVS liegt ebenfalls der Betrachtung von Albers et al. (2020) zugrunde, die trotz Corona-Krise unter dem Titel „Studie zeigt: Die reichsten 50 Prozent werden immer reicher, Arme bleiben arm" mediale Aufmerksamkeit erfuhr (Greive, 2020). Mit Hinzuschätzungen insbesondere im Bereich der Betriebsvermögen und im oberen Vermögensbereich begegnet die Studie einigen Nachteilen des Datensatzes. Gleichwohl zeigt sich inklusive der Hinzuschätzungen, dass der Vermögensanteil der unteren 50 % im Zeitraum bis 2008 gesunken ist und sich seither eher eine steigende bis stabile Entwicklung abzeichnet (Albers et al., 2020, Abbildung 13, 39). Niedrigzinsen und steigende Immobilienpreise können somit für diese Entwicklung beispielsweise eher nicht ursächlich sein, da diese vor allem für den Zeitraum nach der Finanzkrise prägend waren.

4 Forschungsarbeiten des Instituts für Angewandte Wirtschaftsforschung (IAW) für den 6. Armuts- und Reichtumsbericht der Bundesregierung (ARB), die beim zweiten Symposium des ARB vorgestellt wurden, deuten beispielsweise darauf hin, dass der Beschäftigungszuwachs für sich genommen ungleichheitsreduzierend gewirkt hatte und insbesondere Kompositionseffekte eine große Rolle für die Entwicklung ab 2005 gespielt haben. Vgl. Forschungsprojekt „Analyse der Einkommens- und Vermögensverteilung" für den sechsten ARB, Institut für Angewandte Wirtschaftsforschung (IAW), Ifo Institut, München, in Zusammenarbeit mit Martin Biewen, Miriam Sturm, Universität Tübingen, https://www.armuts-und-reichtumsbericht.de/SharedDocs/Downloads/Berichte/zweites-symposium-arb6-praesentation-uni-tuebingen.pdf?__blob=publicationFile&v=4 (26. März 2020).

5 Das zugrundeliegende Nettovermögen besteht aus Finanzvermögen (Bargeld, Bankguthaben und Aktien) zuzüglich realer Vermögen (Häuser, Autos und Grundstücke) und abzüglich der Verbindlichkeiten eines Haushalts.

Abbildung 3
**Entwicklung finanzieller Sorgen**
Anteil der Bevölkerung ab 17 Jahren mit großen Sorgen

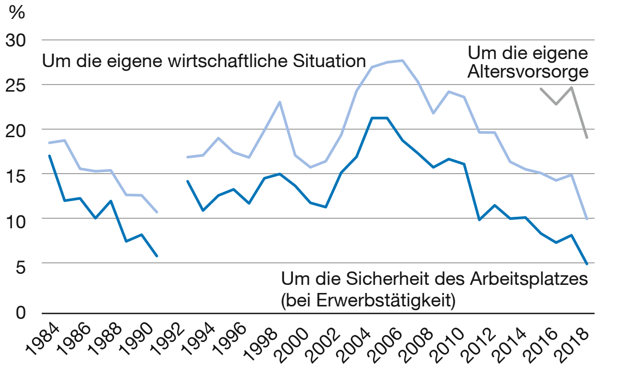

1991: Bis einschließlich 1991 nur Westdeutschland. Anteile auf Basis valider Antworten in den jeweiligen Sorgen-Kategorien. Sorgen zum Thema Altersvorsorge werden erst seit 2015 erhoben.

Quellen: SOEP (2020, v35); eigene Berechnungen.

## Finanzielle Sorgen erreichen Tiefstand – Blick aufs gesellschaftliche Gefüge bleibt jedoch pessimistisch

Die Verteilungsindikatoren deuten darauf hin, dass entgegen weitläufiger Wahrnehmungen auch die unteren Einkommensbereiche von der positiven gesamtwirtschaftlichen Entwicklung der letzten Jahre profitiert haben. Neben der objektiven Entwicklung wird häufig vermutet, dass sich viele Menschen abgehängt fühlen. Insbesondere bei dieser subjektiven Wahrnehmung zeichnen die Befragungsdaten jedoch eine noch stärker positive Entwicklung. In der aktuell verfügbaren SOEP-Erhebung machen sich anteilig so wenige Menschen große Sorgen um ihre eigene wirtschaftliche Situation wie zu keinem Zeitpunkt seit Befragungsbeginn 1984 (vgl. Abbildung 3).

Der Befund impliziert keineswegs, dass es keine finanziellen Sorgen mehr gibt. Hinter den verbleibenden knapp 10 % steht weiterhin die substanzielle Zahl von knapp 7 Mio. Erwachsenen, die ihre finanzielle Lage mit großer Besorgnis beurteilen. Im Jahr 2005 teilten jedoch noch knapp 19 Mio. Erwachsene im SOEP diese Einschätzung.

Eine ähnliche Entwicklung zeigt sich bei den Sorgen um den Arbeitsplatz, wo 2018 beinahe drei Viertel der Erwerbstätigen angaben, dass sie sich keine Sorgen machen; weniger als 5 % machen sich weiterhin große Sorgen um die Sicherheit ihres Arbeitsplatzes. Selbst bezüglich der Altersvorsorge machen sich aktuell weniger Menschen Sorgen als im Jahr 2015, als die Frage im SOEP zum ersten Mal erhoben wurde.

So sehr sich die positiven Einschätzungen mit Befunden zur individuellen Wahrnehmung aus anderen Datenquellen decken, so sehr stehen sie gleichzeitig im Widerspruch zu der Wahrnehmung, dass die Menschen in Deutschland zutiefst verunsichert und von zunehmenden Abstiegssorgen geprägt sind.

Insgesamt zeichnet die Datenlage ein weitgehend positives Bild für die Entwicklung seit 2005 – sowohl bezüglich objektiver Indikatoren von Einkommen und Vermögen als auch bezüglich der subjektiven Wahrnehmung der persönlichen finanziellen Lage. Gleichwohl legen Umfragen regelmäßig nahe, dass der Blick der Bürger auf die gesellschaftliche Lage eher kritisch ausfällt: „Die Bürger empfinden Deutschland als extrem ungerecht", titelte Spiegel Online (Diekmann, 2020) zu Beginn des Jahres 2020 auf Basis einer Civey-Erhebung unter 5.007 Teilnehmern. Gemäß der Umfrage waren 43,9 % der Befragten der Meinung, die Ungleichheit der Einkommen habe in den letzten fünf Jahren eindeutig zugenommen, weitere 28,6 % teilten die Auffassung, sie habe „eher zugenommen", obgleich die oben dargelegte empirische Evidenz für diesen Zeitraum eher für stabile Verteilungsverhältnisse spricht.

Wie sich die weltweite Corona-Pandemie auf die Verteilungsverhältnisse in Deutschland auswirkt, lässt sich bisher nicht abschätzen. Mit Blick auf die ersten Wirtschaftsprognosen ist jedoch anzunehmen, dass sich die Sorgen der Bürger mit dem einschneidenden Ereignis der Corona-Krise mindestens temporär schlagartig verändern dürften. Es bleibt zu hoffen, dass die umfangreichen staatlichen Maßnahmen die Auswirkungen der Krise so gut abfedern, wie es bei der Finanzkrise gelungen ist. Rückblickend lässt sich die Dekade von 2010 bis 2019, mit Rekord-Beschäftigung, solidem Wirtschaftswachstum und stabilen Verteilungsverhältnissen dann sicherlich als ein überaus gutes Jahrzehnt einordnen – wenngleich während dieser Zeit die Debatten eine positive Entwicklung kaum vermuten ließen und viele Menschen diese nur mit Blick auf ihre eigene Situation, nicht aber auf die Gesellschaft, wahrgenommen haben.

Dr. Judith Niehues ist Leiterin der Forschungsgruppe Mikrodaten des Instituts der deutschen Wirtschaft Köln.

Dr. Maximilian Stockhausen ist dort wissenschaftlicher Mitarbeiter im Kompetenzfeld „Öffentliche Finanzen, soziale Sicherung und Verteilung”.


Literatur

Albers T N H Bartels C Schularick M Die Verteilung der Vermögen in Deutschland von 1895 bis 2018 ECONtribute Working Paper 2020
Bundesagentur für Arbeit BA Arbeitslosigkeit im Zeitverlauf 2019
Bundesministerium für Arbeit und Soziales BMAS Lebenslagen n Deutschland Fünfter Armuts-und Reichtumsbericht der Bundesregierung 2020
Deutsche Bundesbank Vermögen und Finanzen privater Haushalte in Deutschland. Ergebnisse der Vermögensbefragung 2017 Monatsberichte 2019 13 44
Diekmann F Spiegel-Umfrage Bürger empfinden Deutschland als extrem ungerecht 2020
Fedorets A Grabka M M Schröder C Seebauer J Lohnungleichheit in Deutschland sinkt DIW Wochenbericht 2020 Nr.7 91 97
Grabka M M Westermeier C Große statistische Unsicherheit beim Anteil der Top-Vermögenden in Deutschland DIW Wochenbericht 2015 Nr.7 123 133
Grabka M M Halbmeier C Vermögensungleichheit in Deutschland bleibt trotz deutlich steigender Nettovermögen anhaltend hoch DIW Wochenbericht 2019 Nr.40 735 745
Greive M Studie zeigt: Die reichsten 50 Prozent werden immer reicher 2020
Niehues J Stockhausen M Ungleichheit(en), ein bekanntes Phänomen? Ifo Schnelldienst 2020 73 2 3 6
Peichl A Die Macht der Zahlen: Ein kritischer Blick auf die Quantifizierung von Ungleichheit ifo Schnelldienst 2020 73 2 6 9
Spannagel, D., K. Molitor (2019), WSI-Verteilungsbericht 2019. Einkommen immer ungleicher verteilt, WSI Report, Nr. 53, Düssseldorf.
Sozio-oekonomisches Panel SOEP Daten für die Jahre 1984-2016 2019
Sozio-oekonomisches Panel SOEP Daten für die Jahre 1984-2017 2020
Statistische Ämter des Bundes und der Länder SAEBL Sozialberichterstattung zur Mindestsicherung 2020
Statistisches Bundesamt Destatis Volkswirtschaftliche Gesamtrechnungen Wichtige Zusammenhänge im Überblick 2019 2020
Stockhausen, M., M. Calderón (2020), IW-Verteilungsreport 2020. Stabile Verhältnisse trotz gewachsener gesellschaftlicher Herausforderungen, IW-Report, Nr. 8, Köln.
